# Oxygen and the Spark of Human Brain Evolution: Complex Interactions of Metabolism and Cortical Expansion across Development and Evolution

**DOI:** 10.1177/10738584221138032

**Published:** 2022-12-08

**Authors:** Andrea I. Luppi, Fernando E. Rosas, MaryAnn P. Noonan, Pedro A. M. Mediano, Morten L. Kringelbach, Robin L. Carhart-Harris, Emmanuel A. Stamatakis, Anthony C. Vernon, Federico E. Turkheimer

**Affiliations:** 1 Department of Clinical Neurosciences and Division of Anaesthesia, School of Clinical Medicine, University of Cambridge, Cambridge, UK; 2 Leverhulme Centre for the Future of Intelligence, University of Cambridge, Cambridge, UK; 3 The Alan Turing Institute, London, UK; 4 Department of Informatics, University of Sussex, Brighton, UK; 5 Centre for Psychedelic Research, Department of Brain Science, Imperial College London, London, UK; 6 Centre for Complexity Science, Imperial College London, London, UK; 7 Centre for Eudaimonia and Human Flourishing, University of Oxford, Oxford, UK; 8 Department of Experimental Psychology, University of Oxford, Oxford, UK; 9 Department of Psychology, University of Cambridge, Cambridge, UK; 10 Department of Psychology, Queen Mary University of London, London, UK; 11 Department of Computing, Imperial College London, London, UK; 12 Center for Music in the Brain, Aarhus University, Aarhus, Denmark; 13 Department of Psychiatry, University of Oxford, Oxford, UK; 14 Psychedelics Division–Neuroscape, Department of Neurology, University of California San Francisco, San Francisco, CA, USA; 15 MRC Centre for Neurodevelopmental Disorders, King’s College London, London, UK; 16 Department of Basic and Clinical Neuroscience, Institute of Psychiatry, Psychology and Neuroscience, King’s College London, London, UK; 17 Department of Neuroimaging, Institute of Psychiatry, Psychology and Neuroscience, King’s College London, London, UK

**Keywords:** oxygen, aerobic glycolysis, cortical expansion, complex systems, transmodal association cortex, social brain, plasticity, evolution, development, metabolism

## Abstract

Scientific theories on the functioning and dysfunction of the human brain require an understanding of its development—before and after birth and through maturation to adulthood—and its evolution. Here we bring together several accounts of human brain evolution by focusing on the central role of oxygen and brain metabolism. We argue that evolutionary expansion of human transmodal association cortices exceeded the capacity of oxygen delivery by the vascular system, which led these brain tissues to rely on nonoxidative glycolysis for additional energy supply. We draw a link between the resulting lower oxygen tension and its effect on cytoarchitecture, which we posit as a key driver of genetic developmental programs for the human brain—favoring lower intracortical myelination and the presence of biosynthetic materials for synapse turnover. Across biological and temporal scales, this protracted capacity for neural plasticity sets the conditions for cognitive flexibility and ongoing learning, supporting complex group dynamics and intergenerational learning that in turn enabled improved nutrition to fuel the metabolic costs of further cortical expansion. Our proposed model delineates explicit mechanistic links among metabolism, molecular and cellular brain heterogeneity, and behavior, which may lead toward a clearer understanding of brain development and its disorders.

## The Explanandum: Human Brain Evolution

**Figure 1.**
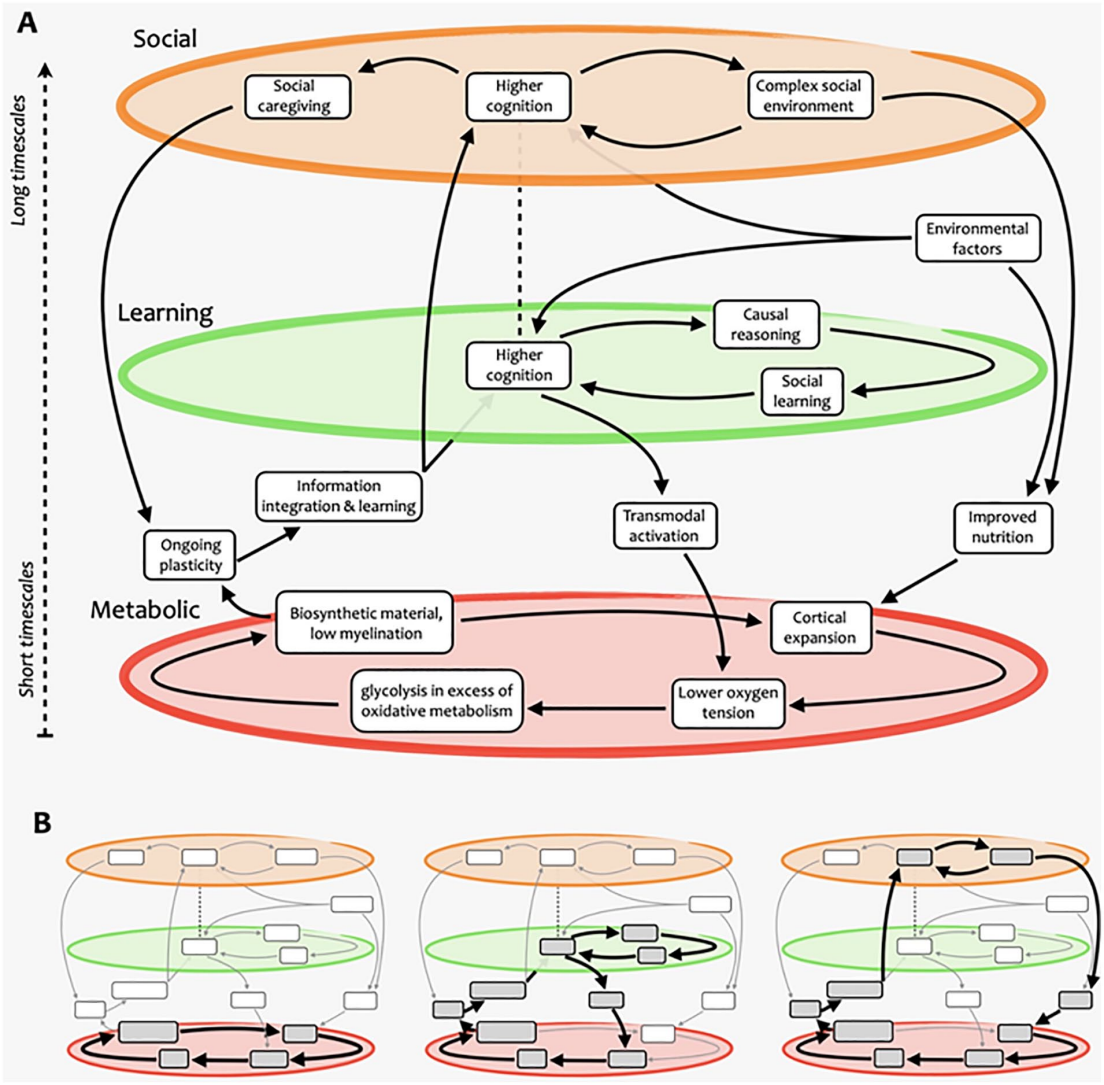
Schematic overview of the complex-systems account of human brain growth and evolution. (A) Our approach presents brain evolution as resulting from multiple nested, mutually reinforcing cycles that span several domains and operate over a variety of different scales—some within the individual, others across generations. (B) Examples of cycles that exist within this complex process. The first part of our presentation focuses on the metabolic cycle (left), and the second part explains how it is embedded in a larger system involving learning and social aspects (center and right), which operate at longer time scales.

What aspects of brain organization are responsible for humans’ sophisticated cognitive capabilities? Since Darwin’s proposal to embed *Homo sapiens* within an evolutionary continuum with the rest of the natural world, neuroscientists and evolutionary biologists have been tasked with providing a neural explanation for the cognitive gap between humans and other species and for the emergence of such differences. Given that cognitive abilities depend on brain structure and function, such explanations have been attempted by using several observations across a range of scales—from the genetic to the metabolic to the behavioral (endo)phenotype. Here, we consider how multiple accounts, operating across vastly different biological and temporal scales, may be brought together to account for the inherently complex phenomenon of human brain evolution (Figure 1) (Turkheimer and others 2021).

The human brain comprises a substantially greater number of neurons than the brains of other great apes: approximately 86 billion neurons for humans, 28 billion for chimpanzees, and 33 billion for gorillas (Herculano-Houzel and Kaas 2011). Additionally, humans surpass other primates in terms of brain-to-body ratio (Silver and Erecinska 1998). However, it has been suggested that these facts might not be in need of special explanations, as the human brain and its associated functional and metabolic characteristics may be understood as the mere allometric expansion of the primate brain, with new functions potentially emerging when scaling involves a pronounced change of proportions (Azevedo and others 2009; Herculano-Houzel 2009; Seymour and others 2016; West and others 1997; White and others 2019), such as changes in the modular structure of the connectome (Changeux and others 2021; Suárez and others 2022).^1^ In effect, the human brain appears to contain as many neuronal and nonneuronal cells as would be expected of a primate brain of its size (Herculano-Houzel 2009)—although recent evidence does indicate the existence of neuronal differences between primates and nonprimate mammals (Banovac and others 2021; Krienen and others 2020).

Going beyond total brain size, however, human brains surpass those of other primates in terms of total volume and total surface area of the neocortex (Donahue and others 2018; Herculano-Houzel and others 2007; Semendeferi and others 2001). Accounting for 20.9% of the total neuron count, the human neocortex outstrips any other mammalian neocortex by >10% (Herculano-Houzel 2012). Crucially, even across the cortex there are important evolutionary differences: cytoarchitectonic and comparative neuroimaging studies have converged on the conclusion that so-called transmodal association cortices in the human brain, where multiple sensory streams from unimodal cortices converge for further processing (Mesulam 1998), are greatly expanded relative to that of other primates (Bruner and others 2017; Glasser and others 2014; Smaers and others 2017; Wei and others 2019; Xu and others 2020). Moreover, this increase is an outlier when compared with the allometric scaling of primate brains (Sherwood and Smaers 2013; Smaers and others 2017).^2^ Therefore, it seems clear that, even in purely physiologic terms, human brains are indeed remarkable and in need of a dedicated explanation.

This endeavor has proven fruitful, delivering a sizable number of comparative accounts of brain evolution that, implicitly or explicitly, aim to explain human cognitive evolution (Box 1). Although each of these explanatory strategies has clear merits, we note that they are not mutually exclusive, as they tend to address distinct parts of the brain’s evolutionary process. We also note that most of the proposed factors exist on a continuum or are shared with other species, making it difficult to rely exclusively on any one of such factors to justify the apparent discontinuity that exists between humans and other species in terms of cognitive capacity. Together, these observations suggest that a suitable answer to the evolution of the human brain and cognition might be best sought, not in any single one of the currently proposed factors, but rather in the synergistic interactions among several. In other words, what is unique about humans might not be any of the proposed factors per se, but rather a specific combination of the interactions among them.

## Part 1: Oxygen and the Human Brain

Here we put forward the core of our proposal, which is built around a simple yet fundamental molecule: oxygen. We capitalize on recent breakthroughs on the role of oxygen in the origin of life (Box 2) to argue that oxygen may also have played a pivotal role in the expansion of the human brain beyond body size—specifically, in the expansion of the human transmodal association cortex. To provide our account of how oxygen shaped cortical expansion, however, we must start by considering the effects of cortical expansion on brain oxygenation.

### Nonallometric Cortical Expansion Favors Lower Oxygen Tension

The brain relies primarily on oxidative metabolism of glucose to extract ATP and fund its extravagant energetic expenditure (Figure 4)—about half of which relates to synaptic transmission (Harris and others 2012), with recent multimodal evidence revealing a positive association between regional glucose metabolism and synaptic density, as measured by synaptic vesicle glycoprotein 2A binding (van Aalst and others 2021). However, as an entirely aerobic organ, the brain does not store glucose or oxygen; hence, it requires a constant supply of both from the blood. Indeed, brain cells make use of the full amount of oxygen delivered, as demonstrated by the fact that although some glucose is lost back into venous circulation in the form of lactate, all oxygen delivered to the parenchyma is disposed only in the form of CO_2_ (Sonnewald 2014). For this reason, brain growth and expansion require increased oxygen and glucose delivery through matching increases of cerebral blood flow and vascular capacity (Seymour and others 2016), which is accompanied by a higher efficiency in the development of brain circuitry (Herculano-Houzel 2011; Levy and Baxter 1996).

Box 1. **Approaches to Explaining Brain Evolution.**

Four broad classes of explanations for brain evolution can be identified (Dunbar and Shultz 2017): relation to body size, opportunity focused, mechanism focused, and constraint focused.

An influential early explanation related to body size was Cope’s rule, which posits a gradual increase in body size over evolutionary time for most population lineages (Rensch 1948) due to the higher selective value of larger individuals when they compete with smaller ones (Kingsolver and Pfennig 2004). This trend is typically attributed to greater strength, resilience, speed, larger weapons, and sensory organs of larger animals (Rensch 1959). Based on this well-established observation, an explanation of brain size increase over evolutionary periods could then be formulated under the assumption that a larger brain is required to govern a larger body (though note that this account does not focus on cognitive abilities). However, recent evidence from fossil scans indicates that the brain-to-body ratio of placental mammals initially decreased, as body growth rate outstripped the rate of brain growth and encephalization only started to actively increase later (Bertrand and others 2022).

Contrasting with this early account that simply posits a larger brain as a by-product of a larger body, opportunity-focused hypotheses propose associations between an increase in brain size and a consequent trait—typically, improvement in some aspect of cognition—which the environment can positively select upon. Depending on the focus, one can distinguish between ecological explanations (Harvey and others 1980), whereby the growth of the primate brain allows a better interaction with demanding environmental conditions, and social explanations, whereby larger brains are better suited to the complexity of primate sociality (Perez-Barberia and others 2007; Shultz and Dunbar 2007).

Mechanism-focused hypotheses are based on the genetics of brain development and focus on identifying uniquely human genes that are implicated in cortical growth (Evans and others 2005; Jayaraman and others 2018; Mekel-Bobrov and others 2005; Mirzaa and others 2014; Montgomery and others 2011; Uddin and others 2008; Wang and others 2008). In addition to expansion of non–protein-coding DNA in humans as compared with nonhuman primates, evidence has been converging on human-specific genes involved in expansion of the fetal cortex and capable of stimulating neurogenesis, such as *ARHGAP11B* (Florio and others 2015; Heide and others 2020; Kalebic and others 2018; Namba and others 2020) and *NOTCH2NL* (Fiddes and others 2018; Florio and others 2018; Suzuki and others 2018). Some of the most recent work in this area uses 3-dimensional organoids to mimic the processes that recapitulate the steps of tissue fate acquisition and morphogenesis during neurodevelopment (Benito-Kwiecinski and others 2020; Chiaradia and Lancaster 2020; Giandomenico and Lancaster 2017; Li and others 2017).^3^ Additionally, several genes have been identified as being responsible for the development of the neocortex because their mutation results in microcephaly and associated linguistic and cognitive deficits, as revealed by convergent evidence in patients and animal models (Jayaraman and others 2018).

Finally, constraint-focused hypotheses are concerned with the physiologic limits to brain tissue expansion (Dunbar and Shultz 2017) and how humans may have succeeded to circumvent them. A relevant constraint to brain expansion is the vulnerability that comes with humans’ very protracted period of immaturity: for many years before reaching adulthood, humans are unable to fend for themselves. Developmental explanations propose that this constraint is overcome thanks to extended periods of parental investment (Barrickman and others 2008) and protection by the social group, with the additional advantage of providing greater opportunity for explorative learning and a key opportunity for transgenerational teaching (Boyd and others 2011).

Another key limiting factor for brain size is the energetic cost of its neuronal mass. In humans, although the brain represents only 2% of total body mass, it consumes an astonishing 20% to 25% of the total body energy budget; in comparison, other primates dedicate approximately 8% to 10% of their metabolism to brain function, and most other mammalian species use only 3% to 5% (Attwell and others 2010; Leonard and others 2003; Magistretti and Allaman 2013). Such high energetic demands clearly place bounds on brain tissue growth (DeCasien and others 2017; Fonseca-Azevedo and Herculano-Houzel 2012; Herculano-Houzel 2012; Isler and van Schaik 2006; Kuzawa and others 2014; Kverkova and others 2018; Navarrete and others 2011; Pontzer and others 2016; Sukhum and others 2016). According to the “expensive tissue” hypothesis, the high energetic requirements of neurons and limited caloric yield of raw food impose a trade-off between body size and brain size (i.e., neuron count), which is still limiting the brain size of today’s great apes; however, our ancestors overcame this limitation and achieved the capacity for greater total energy expenditure than other apes (Pontzer and others 2016). This metabolic acceleration may have in turn unlocked the potential for greater neuronal count, achieved by shifting to more energy-dense foods through frugivory, and when *Homo erectus* started processing and cooking food, thereby extracting more energy in considerably less time (Fonseca-Azevedo and Herculano-Houzel 2012). In addition, cooking offset the body’s energetic requirements by a corresponding reduction of the requirements of other tissues, for example, by reduction of the size of the gut thanks to increased digestibility of cooked food (Aiello and Wheeler 1995; Huang and others 2018). Cooking is also an excellent example of skill acquired from transgenerational learning, highlighting the possible interdependencies among different accounts.

Box 2. **Oxygen in the Earth’s Geochemistry and the Evolution of Life.**

**Figure 2.**
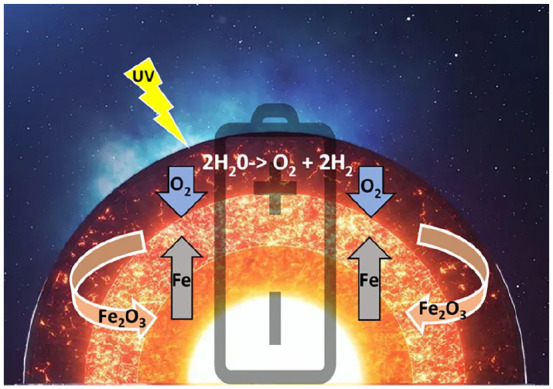
Oxygen-driven energetic fluxes of the earth. Oxygen (O_2_), a strong electron acceptor, was likely present in the earth’s atmosphere from the beginning due to its separation from aqueous vapor in the atmosphere by ultraviolet radiation from the sun. Iron (Fe) and iron alloys in the earth’s inner and outer cores are good electron donors. This imbalance creates an electron flux toward the crust that does not reach equilibrium due to the magmatic fluxes at the bottom of the oceans that replenish the oxidized iron (Fe_2_O_3_) on the earth’s surface. This electron flux from solar emanations dominates the earth’s biochemistry at various levels on its surface, from the depth of the oceans (anoxic environments) to the continents (oxidative environments), generating availability of bioenergy that can be used for growth by both organisms and organs.

**Figure 3.**
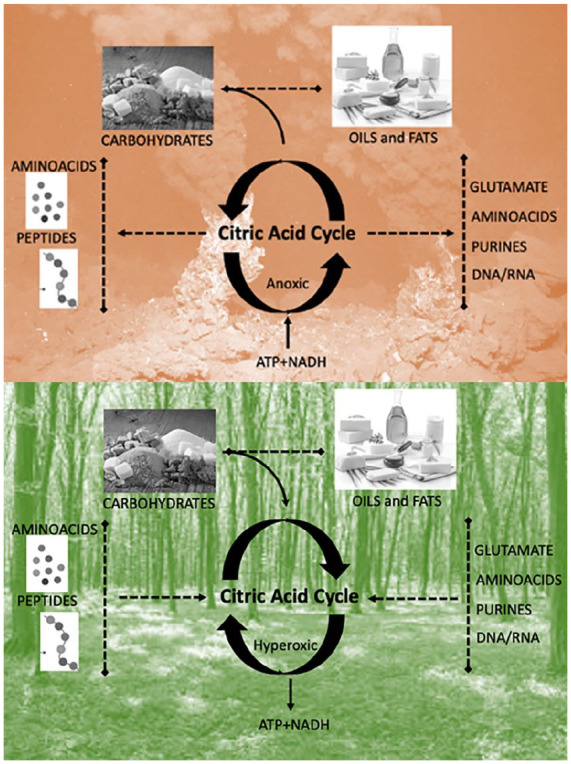
Opposite directions of the citric acid cycle depending on environmental conditions. Top: In anoxic environments with high temperatures, such as the ones found in deep sea alkaline vents, the citric acid cycle functions in an anticlockwise manner, fixating hydrogen on carbon chains and hence producing the basic constituents of life—including carbohydrates, amino acids, and fats. Bottom: In the oxidative environments with low temperatures, as on the continents, the citric acid cycle functions in a clockwise fashion, oxidizing carbohydrates, proteins, and fats. This fixates the excess O_2_ in the atmosphere into CO_2_ and releases of energy in the form of ATP. This can be seen as a primordial form of the citric cycle, also known as the Krebs cycle or tricarboxylic acid cycle, which is the core motor of all metabolic reactions.

Modern planetary science has shown how the earth’s first biochemistry was shaped by the interaction of the energy of solar radiation and both the gases in the earth’s atmosphere and the metals in its core. Oxygen is the most abundant element in the earth’s crust (Dole 1965), and it was probably present in the crust from very early on.^4^ Oxygen is an ideal electron acceptor (Allred and Rochow 1958), being the second-most electronegative element. Yet, the earth’s core is composed mostly of iron (inner core) and iron alloys (outer core), which are good electron donors. This creates a strong imbalance between powerful oxidative forces on the crust and reducing fluxes from the core that never fully equilibrate—given that magmatic fluxes in the oceans’ depths keep replenishing the oxidized metals of the crust, which effectively creates conditions where the earth is analogous to a giant battery (Figure 2)—with an oxygen-rich pole on the surface and an iron-rich pole in the core (Smith and Morowitz 2016). The interplay of iron and oxygen may be at the very core of the emergence of life on our planet (Wade and others 2021). It has been postulated that life may have started during the Hadean period (~4 billion years ago) at the juncture between these opposed poles—in alkaline vents in the bottom of the ocean that acted as electrochemical flow reactors (Braakman and Smith 2014; Lane 2017; Miller and Bada 1988; Sojo and others 2016) (Figure 3, top). This model portrays the basic biochemistry of life as a consequence of the energy imbalances in the earth’s early geochemistry, with living creatures serving as relaxation channels to reduce the free energy gradients of our planet (Smith and Morowitz 2016)—making organic life not an “unlikely” but perhaps almost “necessary” product of biochemical potentials of the earth’s inorganic milieu. The resulting abundance of O_2_ in the atmosphere likely promoted the survival of organisms capable of tolerating the toxicity associated with oxygen (damaging free radical reactions) and favored cellular mechanisms that could convert the gas safely and use it to extract energy (Figure 3, bottom). With time, oxidative phosphorylation (oxidization of glucose to extract energy in the form of ATP) developed into aerobic respiration, with the symbiotic merger of cells with the once free-living α-proteobacteria that then became the mitochondrion (Gray and others 2001). In turn, thanks to its comparatively high ATP yield, aerobic respiration has been argued to have favored the evolutionary transition from unicellular to multicellular organisms (Pfeiffer and others 2001). In bigger vertebrates, aerobic respiration then beckoned the evolution of organ systems that were dedicated to the absorption and transportation of O_2_—that is, the evolution of respiratory and cardiovascular systems (Bailey 2019; Costa and others 2014). Relationships between body mass and oxygen metabolism have been found in mammals (Dlugosz and others 2013), gastropods (Marsden and others 2012), and insects, where experimental manipulation indicated that growth and size depend on the relative scarcity or abundance of oxygen (Harrison and others 2010). It has also been argued that an increase in the abundance of atmospheric oxygen may have contributed to the evolution of larger mammals by facilitating placental mammal reproduction (Falkowski and others 2005).

**Figure 4.**
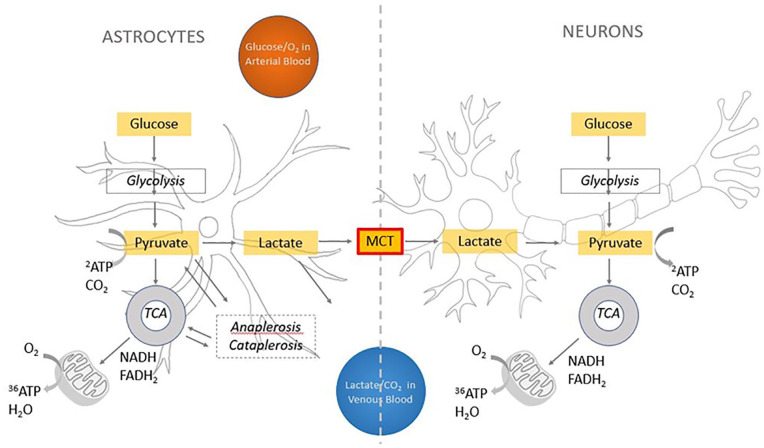
Energy metabolism in the brain. The main source of energy of the brain is the oxidative metabolism of glucose. Once it is transported into the brain, glucose goes through glycolysis, where it is metabolized in pyruvate with a small production of ATP (two ATP molecules for each glucose molecule). Pyruvate then enters the tricarboxylic acid (TCA) cycle (Figure 3), which besides a small production of energy, produces NADH and FADH2 that feed the mitochondrial electron transport chain. Electrons are passed from protein to protein in this chain, where oxygen comes into play as the end electron acceptor, picking up two hydrogen ions and becoming water. This process produces a large quantity of ATP (30–38 molecules). According to Pellerin and Magistretti (1994), pyruvate in astrocytes is turned into lactate that is transported by monocarboxylate transporter (MCT) into neurons and transformed back into pyruvate to feed neuronal oxidative metabolism. According to the model proposed by Sonnewald (2014), a generalized excess in glycolysis in the brain—the ratio of oxygen metabolism to glucose metabolism is lower than the expected ratio of 6—may be due to the use of pyruvate, in astrocytes only, for the two complementary processes of anaplerosis and cataplerosis, which produce intermediates of the TCA cycle as well as glutamate, GABA, and aspartate. The process is not known in detail, but the net flux is thought not to feed into oxidative glycolysis but instead to turn into lactate that is eliminated by vascular or lymphatic circulation. In our proposal, we note that in the neocortex MCT density is far reduced, supporting the idea that less oxygen may be available for its oxidation in neurons. Anaplerosis is known to be present in the early developing brain because of the need of carbon skeletons as well as de novo neurotransmitters. However, its requirement in the adult brain is far lower and, in the authors’ opinion, does not suffice to justify the far excessive glycolysis in the neocortex.

Although allometric relations are generally stable within each taxon, the upward divergence of the human brain with respect to other primates suggests that the oxygen tension (i.e., partial pressure of oxygen, reflecting the balance between local oxygen delivery and consumption) may be lower in the extra tissue of the evolutionarily expanded cortical regions. This is suggested by the following evidence:

- Allometric scaling relations are determined by the capacity of vascular systems to deliver nutrients (West and others 1997).
- There is a strong relationship between oxidative metabolism and microvascular density (Keller and others 2011; Weber and others 2008), with nonprimary cortical areas being similar in microvascular architecture, having lower density than primary visual, somatosensory, or auditory areas (Schmid and others 2019).
- Supporting this evidence from postmortem and animal studies, a recent high-resolution atlas of the human brain’s venous vasculature obtained from in vivo 7-T MRI indicates that the highest levels of vascular density are found in the insula and primary visual and auditory cortices (Huck and others 2019).^5^ Hence, given that it does not appear that vascular efficiency is increased in the human association cortex, we argue that circulation should not able to provide the same amount of oxygen for unit volume for the tissue exceeding the allometric ratio.^6^

Thus, evolutionarily expanded cortical regions—which largely coincide with transmodal association cortices—should be characterized by lower oxygen tension than the primary sensory and motor cortices. This lower oxygen tension may be expected to result from the fact that these expanded regions use all available oxygen; thus, extra tissue cannot rely on oxidative phosphorylation to extract energy from the remaining glucose provision—which exceeds oxygen availability. Consequently, association cortices should rely on the metabolism of glucose outside of oxidative phosphorylation (glycolysis in excess of oxygen availability [GEOA]) by fermenting glucose into lactate,^7^ as demonstrated by empirical evidence (Glasser and others 2014; Goyal and others 2014; Vaishnavi and others 2010).^8^

By itself, the observation of greater glucose utilization than that predicted by oxygen consumption does not necessitate that there is not enough oxygen available, because glycolysis may occur for other reasons than oxygen being insufficient for full metabolism of glucose via oxidative phosphorylation. Therefore, the claim of insufficient oxygenation is rather an inference that we draw, based on the evidence that oxygen is fully used up in the brain (Sonnewald 2014) and the argument from vascular efficiency outlined earlier.^9^ Therefore, multiple lines of evidence lead us to hypothesize that the elevated GEOA observed in association cortices may be at least in part due to diminished oxygen tension, which we related to these regions’ expansion.

## Differences in Oxygen Tension Contribute to Shaping Cytoarchitecture across the Cortical Hierarchy

This regional difference in oxygen tension may contribute to explaining how one of the most defining features of the human cortex comes to be established: namely, its hierarchical organization, recapitulating the progressive feedforward integration of information from sensory to association cortices, with the latter coinciding with evolutionarily expanded regions.^10^ In addition to the known regional differences in gene expression across the cortical hierarchy (Burt and others 2018; Hansen and others 2021), we propose that hierarchically organized regional differentiation may be facilitated by differences in regional metabolism regulating cytoarchitecture. Specifically, oxygen tension has been shown to regulate proliferation, differentiation, and maturity of glia and neurons from pluripotent stem cells in the developing brain (Simon and Keith 2008). On one end, relatively higher oxygen tension seems to favor the growth of neurons and oligodendrocytes, with a consequent increase in intracortical myelination, which is most pronounced at lower levels of the human cortical hierarchy (Burt and others 2018). Indeed, oligodendrocytes rely on oxidative metabolism at a greater rate than other cells in the brain, due to the metabolic costs of myelin maintenance (Connor and Menzies 1996). The same authors also note that for their heavy reliance on oxygenation, oligodendrocytes are closely tied to vascularization—which evidence indicates is less efficient in association cortices. In contrast, relatively lower oxygen tension seems to rebalance the cell milieu away from myelinating oligodendrocytes and toward astrocytes (Xie and others 2014; Yang and others 2018), which supports glucose transport and metabolism and whose regional distribution is higher in association cortices (Pellerin and Magistretti 1994). Using in vivo imaging in the developing zebrafish, Yang and others (2018) found that reduced oxygen tension suppressed oligodendrocyte progenitor cell migration and resulted in thinner myelin sheaths. Additionally, Xie and others (2014) studied stem cells and through gain- and loss-of-function studies demonstrated that oxygen tension contributes to determining whether neural progenitors develop into neurons or glia. Furthermore, via stimulation of mitochondria metabolism in human neurons, Iwata and others (2021) managed to control accelerated maturation. Thus, we contend that by contributing to regulating the type of cells that develop in a given region, oxygen tension and metabolism may act as a key contributors to establishing higher levels of GEOA at the top of the cortical hierarchy and higher myelination at the opposite (lower) end of the hierarchy.^11^

### Glycolytic Metabolism Promotes Synapse Growth and Plasticity

GEOA and myelination have important consequences for brain function, specifically in terms of how they regulate synaptic plasticity. Mechanistically, the capacity for ongoing learning throughout development and into adulthood—a key feature of human cognition—needs to be supported by persistent synaptic plasticity and synaptic turnover. This, in turn, requires constant availability of biosynthetic material to generate new synapses and maintain existing ones. Glycolytic metabolism is well suited to satisfy this need, because in addition to generating ATP, the glycolytic process produces glycolytic intermediates (amino acids, lipids) that can be used as building blocks to support biosynthesis (Bauernfeind and others 2014; Vander Heiden and others 2009): Besides contributing to the production of neurotransmitters glutamate/GABA, they are required for the production of carbon skeletons that are used for neuronal growth/adaptation. Indeed, the regional rate of GEOA correlates with transcription of genes pertaining to synapse formation and growth (Goyal and others 2014). Additionally, the uptake of glutamate (involved in long-term potentiation, depression, and synaptic plasticity) into astrocytes is believed to rely on ATP produced via glycolytic metabolism (Horvat and others 2021; Pellerin and Magistretti 1994), providing a further link between glycolysis and plasticity. Prefrontal and associative cortices present distinct mRNA profiles that are enriched in the synaptic, dendrite, and somatodendritic compartments and the perikaryon and neuron projection terminus components (Petanjek and others 2011; Wei and others 2019). Postmortem tissue analyses (Jacobs and others 2001) and in vivo imaging (Finnema and others 2016; Shafee and others 2015) have demonstrated that prefrontal and association cortices exhibit the highest synaptic density for the whole of the neocortex and the lowest myelin density.

Myelination has been shown to inhibit synaptic growth, both through signalling and by acting as a mechanical barrier (Fletcher and others 2021). Intracortical myelination has also been shown to reduce spine turnover and LTP in the cortex (Akbik and others 2012; Boghdadi and others 2017; Schwab and Strittmatter 2014). Corroborating these observations, García-Cabezas and others (2017) examined the expression of plasticity and “stability” markers across different cortical types in the prefrontal cortex of adult nonhuman primates, reporting that markers of plasticity (e.g., calcium/calmodulin-dependent protein kinase II) are highest in higher-order cortices, whereas intracortical myelination is lowest.

From a developmental perspective, the adult brain emerges as the result of two separate phases of neurodevelopment. In the first prenatal phase, modulation of brain growth arises largely from neurogenesis. GEOA occurs in rapidly dividing progenitor cells in the embryonic brain, such as the neural progenitors that are especially abundant in humans (Namba and others 2021). The postnatal period instead involves a critical period of growth largely driven by experience-dependent formation of neuronal connections, which appears to involve neuronal arborization and synaptogenesis but also pruning, gliogenesis, and myelination. However, these processes are not uniform across the cortex: in particular, association cortices of the human brain appear to be characterized by the persistence of these developmental features and processes into adulthood—technically known as “paedomorphosis,” the persistence of juvenile traits into adulthood but often also referred to as “neoteny”^12^ in the literature (Somel and others 2009; Somel and others 2011; Vaishnavi and others 2010).^13^ Indeed, GEOA correlates with “cortical neoteny” (Goyal and others 2014), whose manifestations include the persistence of gene expression patterns associated with synapse formation and turnover in association cortices (Liu and others 2012). Importantly, new evidence points to metabolism, via mitochondrial maturation, as controlling the timing of brain development: oxidative phosphorylation is less prevalent and mitochondrial development slower in human cortical neurons as compared with mouse neurons (Iwata and others 2021).

Temporally, converging neuroimaging and meta-analytic evidence indicates that GEOA is highest during the same periods of development when synaptic growth is fastest: it accounts for >90% of the glucose consumed in the preterm infant brain (Goyal and others 2014; Vaishnavi and others 2010), decreasing to 35% in newborns (Settergren and others 1976), and then peaks again at ~5 years of age when the rate of synaptic growth is highest, before reaching adult levels at ~10% of total expenditure (Boyle and others 1994; Goyal and others 2014; Raichle and others 1970). Finally, GEOA declines with aging (Goyal and others 2017).

Spatially, the metabolic switch from the initial prevalence of GEOA to oxidative phosphorylation occurs earlier in primary cortices (at the bottom of the cortical hierarchy) than in transmodal association cortices (at the top of the hierarchy), which still retain high levels of GEOA in adulthood—reflecting the asynchronous nature of cortical neurodevelopment (Bauernfeind and others 2014; Jacobs and others 2001). Nearly 25% of resting glucose consumption is nonoxidative in the medial prefrontal gyrus, whereas in nonassociation cortices glucose oxidation is virtually the sole metabolic support (Glasser and others 2014; Goyal and others 2014; Vaishnavi and others 2010). Transmodal association cortices are also those with the longest maturation times, relative to other regions of the human brain (Gogtay and others 2004) and to other primates: the synaptic density of the human prefrontal cortex peaks around 4 to 5 years of age, whereas for macaques and chimpanzees, this region already achieves peak density within the first year of life (Liu and others 2012). Thus, even during adulthood, GEOA may support continued synapse formation and plasticity.

Evolutionarily, the human brain itself appears to be especially plastic, more so than the brains of nonhuman primates. Morphometric techniques have revealed an elevated level of anatomic fluctuating asymmetry in the human brain as compared with chimpanzees (Gomez-Robles and others 2013). Anatomic fluctuating asymmetry reflects nondirectional departures from the expected bilateral symmetry and is considered a marker of developmental instability; in this context, it suggests that a more substantial and sustained developmental plasticity takes place in human brains (Gomez-Robles and others 2013). This evidence corroborates anatomic and molecular findings from neuroscience and anthropology that led Sherwood and Gomez-Robles (2017) to conclude that the human brain is evolutionarily specialized for an extraordinary degree of plasticity. This overall plasticity does not appear to be uniformly distributed in the human brain either: comparing anatomic scans of nearly 3000 individuals, Reardon and colleagues (2018) considered variations in brain size, finding that larger human brains are preferentially expanded in regions of the transmodal association cortex that coincide with regions of high evolutionary and developmental expansion (Hill and others 2010) and that are enriched for synaptic genes and display elevated glucose uptake.

**Figure 5.**
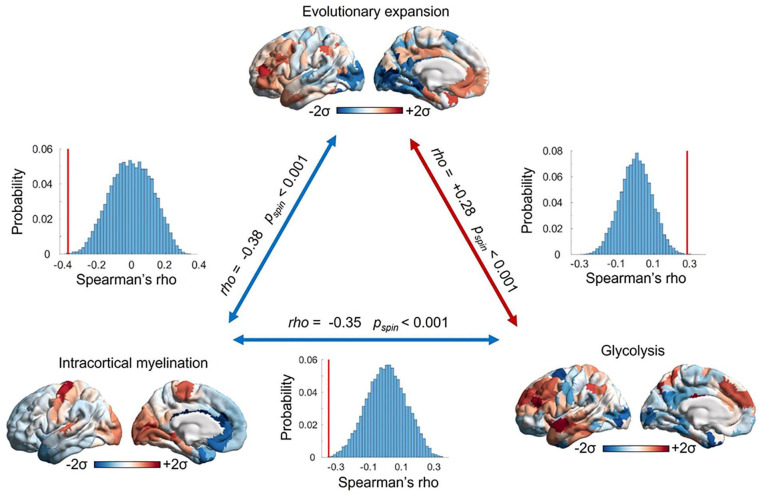
The most evolutionarily expanded cortical areas exhibit low intracortical myelination and elevated glycolysis exceeding oxygen availability. This correspondence is especially evident in the lateral prefrontal and temporal cortices. The opposite pattern can be observed in primary visual and somatomotor regions, which reside at the opposite end of the cortical hierarchy and are high in myelination (Burt and others 2018) but low in both glycolysis (Vaishnavi and others 2010) and evolutionary expansion from the macaque brain (Hill and others 2010). Together, these observations support the idea that evolutionary expansion may be related to high glycolysis in excess of oxygen availability and low intracortical myelination, given their roles in facilitating and inhibiting plasticity, respectively. The significance of correlations is assessed against 10,000 spin-based null models that preserve each map’s spatial autocorrelation, thus ensuring that correlations (red bars) exceed the values that would be expected from spatial autocorrelation alone (histograms; Alexander-Bloch and others 2018; Markello and Misic 2021).

In effect, the regions of the transmodal association cortex exhibiting the highest rates of GEOA are also among the most evolutionarily expanded, and they are characterized by the lowest levels of intracortical myelination—whereas the opposite is true for the sensory and motor regions at the opposite end of the brain’s “archetypal axis” (Sydnor and others 2021) (Figure 5). The adult human brain relies on GEOA for a greater proportion of total resting glucose metabolism than other Old World primates, which also exhibit less pronounced regional differences in metabolism (Bauernfeind and others 2014).

**Figure 6.**
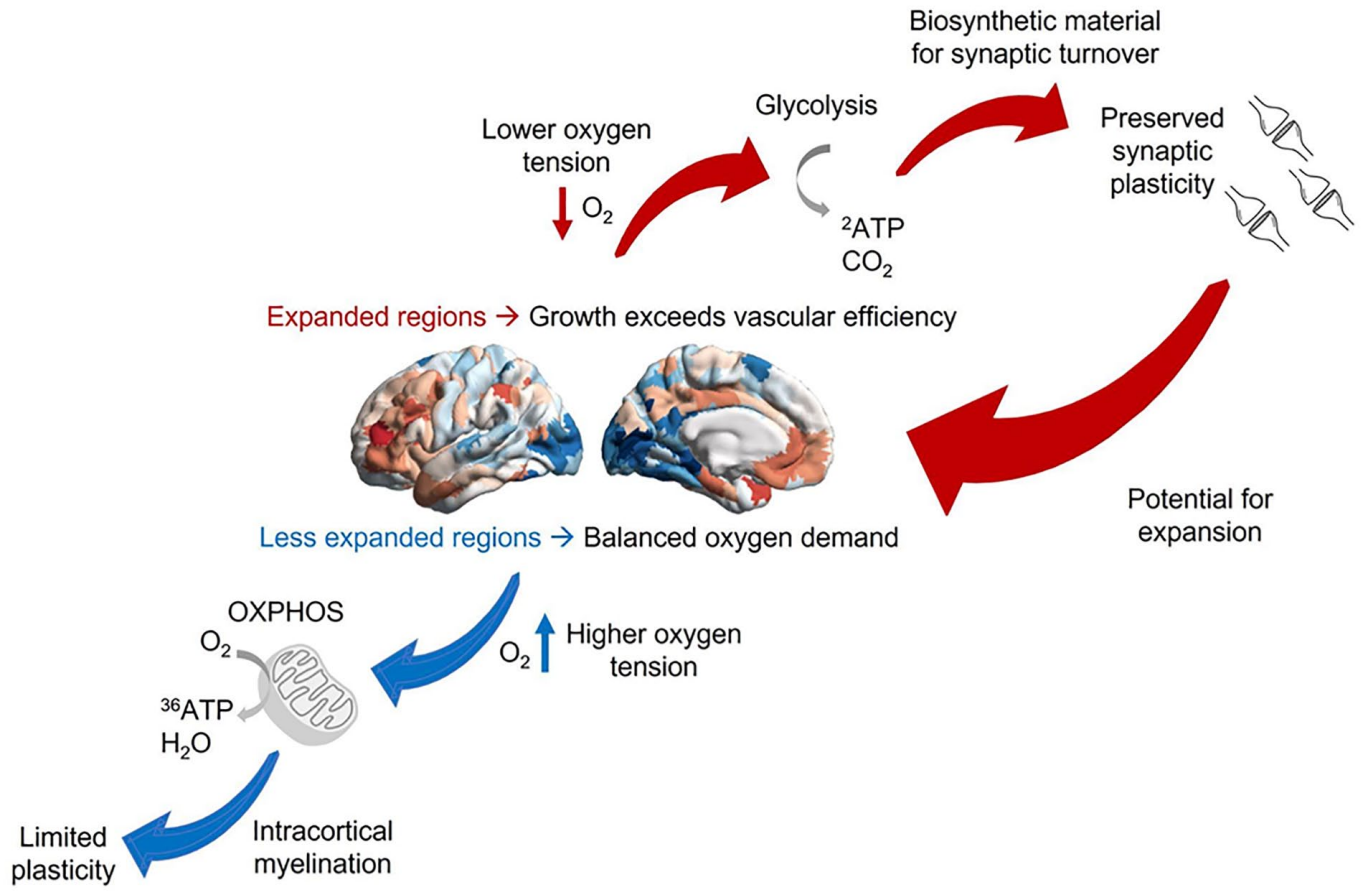
Linking oxygen tension and cortical expansion via metabolism and plasticity. According to this account, less expanded sensory and motor regions have higher oxygen tension and can rely on oxidative metabolism, which promotes intracortical myelination and inhibits plasticity. In contrast, the expansion of human association cortices exceeds the allometric capacity of oxygen delivery by the brain’s vascular system, resulting in lower oxygen tension and requiring increased glycolysis in excess of oxygen availability (GEOA) to compensate for insufficient oxidative metabolism. GEOA in turn contributes to synaptic plasticity by producing the required biosynthetic material, thereby providing the potential for further expansion. As outlined in Part 2, under suitable environmental circumstances, this process can become a cycle, with plasticity and growth promoted by GEOA contributing to further expansion across multiple time scales. Note that at both ends of the hierarchy, metabolic differences also interact with differences in gene expression in contributing to cytoarchitectonic differences.

In summary, the evidence presented in the aforementioned sections provides the grounds for the two tenets of our proposal (right-hand side of Figure 1):

Tenet 1: We propose that the excess growth of human association cortices exceeds the allometric capacity of oxygen delivery by the brain’s vascular system, potentially resulting in lower oxygen tension (partial pressure of oxygen in the blood) that may be insufficient to support full oxidative metabolism of glucose, thereby requiring compensation via increased GEOA. This part of our account seeks to explain why higher levels of GEOA are observed in association cortices rather than elsewhere.
Tenet 2: Oxygen tension is proposed to be a key driver of cellular differentiation and maturation trajectories of different human cortices throughout development. A high oxidative metabolism in cerebellar, primary, and secondary cortices promotes myelination, which leads to efficient but rigid networks (e.g., less plastic). In contrast, lower oxygen tension in association cortices may lead to lower intracortical myelination, favoring higher rates of GEOA, which in turn promotes a type of cellular milieu more favorable to plasticity. As a result, our proposal accounts for the observation that association cortices even at maturation are rich in synapses and still capable of supporting ongoing synapse formation and cortical growth (Figure 6).

## Part 2: From Cortical Expansion to Cognitive Evolution

The arguments presented so far can be summarized as follows: by exceeding vascular efficiency, 1) cortical expansion may have shaped the distribution of oxygen tension in the brain, and 2) regional differences in oxygen tension and GEOA can lead to nonuniform patterns of myelination versus plasticity across the cortex, leading toward a disproportionate expansion of specific cortical regions. In other words, we have identified two components of a self-reinforcing loop, each poised to promote the other.

This last observation raises two key questions: 1) Are these disproportionately expanded regions, rich in GEOA, a suitable candidate for explaining humans’ sophisticated cognitive capabilities? 2) If so, then what is a plausible account explaining how this self-reinforcing loop became and remained viable so that expansion was selected for?

The second part of our account addresses each of these questions in turn. Our proposed explanation lies on identifying additional nested self-reinforcing feedback loops, which involve various biological and social aspects. A core idea in our proposal is that the social environment has the potential for becoming continuously more complex, presenting an “arms race” for individuals in which improvements in affective-cognitive abilities can provide progressively more benefits. We then outline a potential path to “pay the energetic bill” for the metabolic expenditure of evolutionarily expanded regions, based on these regions’ involvement in social cognition.

### Benefits and Costs of Cortical Expansion

One plausible candidate to explain how evolution resulted in cortical expansion is the additional cognitive capacities made possible by expanded association cortices and their elevated, persistent synaptic plasticity. We argue that by providing a substrate to support flexible learning, from one’s own experience and in terms of social learning (Boyd and others 2011), brain plasticity makes it possible to survive and thrive in a wide range of complex environments—for example, those characterized by rapid climatic fluctuations, which may have been a driver of hominin evolution by requiring flexible foraging strategies (Potts and others 2020).

Evolutionarily expanded transmodal association cortices are located at the anatomic confluence of multiple distinct information streams (Mesulam 1988), being placed at the top of the cortical hierarchy—maximally distant from primary sensorimotor cortices—and associated with high-level cognition across a variety of neuroimaging tasks (Fox and others 2020; Margulies and others 2016; Sydnor and others 2021). Recent evidence further indicates that the most evolutionarily expanded regions of the human cortex are especially well suited to support integrative processes because they are the best suited to exploit “synergistic” information: the additional information that one gains by combining multiple sources of information, over and above the sum of their individual contributions (Luppi and others 2022; Mediano and others 2021; Timme and others 2014). Intriguingly, recent evidence indicates that the human brain exhibits a higher proportion of synergistic interactions among its regions than the brains of macaques (Luppi and others 2022). Crucially, postmortem transcriptomic evidence and in vivo PET imaging converge to indicate that regional synergy also correlates with regional synaptic density and prevalence of GEOA (Luppi and others 2022). Thus, evolutionarily expanded regions of the human brain that are high in GEOA also coincide with synapse-dense regions that have the highest capacity to leverage synergistic information. Being endowed with the capacity for ongoing plasticity and little myelination—which our proposal attributes at least in part to the combination of metabolic differences and complementary differences in gene expression—are two aspects that could contribute to making these regions ideally poised to provide the human brain with the flexibility required to master a variety of challenges by leveraging the synergy among different information streams.

However, plasticity is not without costs (DeWitt and others 1998). In the brain, evolutionarily expanded regions are disproportionately expensive in terms of energy, owing to their reliance on GEOA for their metabolism. The reason is that GEOA is an inefficient way of extracting energy from glucose: it produces only 2 ATP molecules from each molecule of glucose, whereas oxidative metabolism would produce 30 (Berg and others 2002). This suggests that the process of cortical expansion leading to GEOA should have happened in a context where less efficient methods to extract energy are still viable—whether because sources of energy were not overly scarce, or because cooking made it possible to extract more calories from food (Herculano-Houzel 2016).

Building on this, it is reasonable to argue that such a process would critically rely on a scenario where the organism has access to plenty of energy (e.g., via a suitable diet). Over the course of human evolution, diverse sources of nutrients have featured in the diet of humans (Wrangham 2013), which may have influenced brain expansion by providing the required energetic or biosynthetic supply (Namba and others 2021). As an example of the potential relevance of diet, frugivory—nutrition based on fruit rather than leaves, which is therefore higher in energy-rich sugars—has been found to correlate with brain size across species (DeCasien and others 2017), possibly by providing support for higher energetic turnover during development and beyond while reducing the amount of resources required for digestion (Isler and van Schaik 2009; Pontzer and others 2016).

However, even if the environment provided the means for extracting sufficiently high amounts of energy, we still face the question of how the same environment selected for expansion to occur. We address this question by building on the “social brain hypothesis” literature (Dunbar 1998, 2003, 2009), which argues that evolutionarily expanded regions of the human brain appear to subserve key functions for successful social interaction, and we outline how this may have provided a path to “pay the energetic bill” for these regions’ metabolic expenditure.^14^

## Cortical Expansion and Complex Social Dynamics: A Self-Reinforcing Feedback Loop

**Figure 7.**
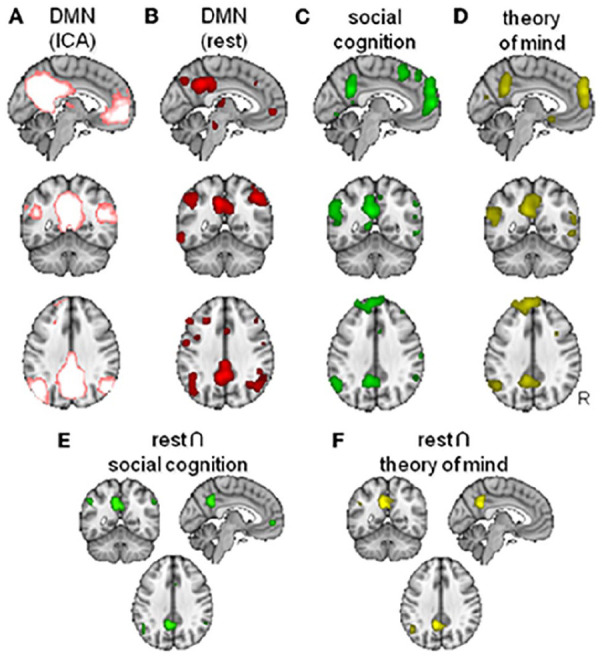
Overlap between the “default mode” network (DMN) and the “social brain” (figure from Mars and others 2012). (A) DMN as defined from model-free Independent Component Analysis (ICA). (B) DMN as defined from task-free “rest” activation. (C) Contrast identifying regions involved in social cognition. (D) Contrast identifying regions involved in “theory of mind” tasks. (E) Conjunction map for the overlap between rest and social cognition. (F) Conjunction map for the overlap between rest and theory of mind.

Evolutionarily expanded regions exhibit remarkable overlap with the so-called social brain—that is, the set of regions pertaining to understanding, predicting, and manipulating others’ mental states (“mentalizing” or “theory of mind”), a skill that is argued to be particularly sophisticated in humans versus other primates (Saxe 2006; Lockwood and others 2020; Tso and others 2018). The regions supporting mentalizing include temporoparietal junction and medial prefrontal cortices, which are also part of the brain’s high-synergy “default mode” network (DMN; Dohmatob and others 2020; Mars and others 2012; Raichle and others 2001) (Figure 7). Changes in functional connectivity within the DMN and structural changes in white matter tracts connecting DMN nodes have also been shown to correlate with the size of an individual’s social network (Noonan and others 2018). Regions belonging to the DMN have been shown to exhibit correlated activity across different individuals observing the same movie (Yeshurun and others 2021). Crucially, however, this similarity of DMN activity is dependent on the social distance among the individuals in question, being strongest among friends, weaker in friends of friends, and virtually absent beyond that (Yeshurun and others 2021). Neurons have even been identified in dorsomedial portions of the DMN that not only monitor another’s action to guide one’s own but also predict an opponent’s behavior (Haroush and Williams 2015). These observations suggest that DMN regions, which are evolutionarily expanded in humans, may support humans’ ability to predict the mental states of their closed associates in response to a given event, which is a key requirement for successful social coordination. Indeed, even when matched in terms of general cognitive skills, human children display far greater skill at social cognition than chimpanzees or orangutans (Herrmann and others 2007).

Building on this intriguing overlap, the “social brain hypothesis”^15^ posits that the necessity to navigate complex social dynamics within the group and with outgroup conspecifics (Ashton and others 2020) would have exerted evolutionary pressures that favored the ability to interpret, predict, leverage, and manipulate others’ behavior—and the consequent growth of associated cortical structures, according to the theory (Byrne and Whiten 1989; Dunbar 1998, 2003, 2009; Humphrey 1976; Whiten and Byrne 1997). Dealing with such interactions extends the range and diversity of cognitive abilities that are required from a given individual, thereby contributing to the development of progressively more domain-general and flexible cognitive capacities (Humphrey 1976). In turn, as proponents of the social brain hypothesis have long argued, there is a deep analogy between inferring others’ mental states as causes of their actions, and inferring unobserved causes of observed effects in the environment; similarly, predicting others’ behavior based on their inferred mental states involves the same kinds of processes required for more general causal reasoning (Molapour and others 2021). Additionally, cooperation allows social animals such as primates to engage into more elaborate strategies for gathering food. Interestingly, this fits well with the so-called clever foraging hypothesis (Striedter 2004), which posits that highly encephalized species tend to forage or hunt strategically (i.e., leveraging the target’s properties or habits), while less encephalized species tend to gather food opportunistically.

Another key opportunity of living in groups is the possibility to learn from conspecifics over developmental rather than evolutionary time scales (Molapour and others 2021). Living with a group of conspecifics offers the opportunity for cumulative intergenerational learning—at which humans are especially successful thanks to language—and to learn from others’ mistakes, rather than one’s own, while benefiting from their successes (Whiten 2005). As Boyd and others (2011) argued, the capacity to learn from one another “enables humans to gradually accumulate information across generations and develop well-adapted tools, beliefs, and practices that no individual could invent on their own,” representing an essential element of humans’ ecologic success. This kind of learning is ongoing rather than a one-off, both across generations and during one’s lifetime—thereby benefiting from the ongoing plasticity of transmodal association cortices.

Additionally, neural plasticity seems required for supporting behavioral flexibility in the form of tool use, which inherently entails an adaptation of one’s behavior and an integration of cognition, body, and tool (Bruner 2021; Bruner and Gleeson 2019; Bruner and others 2022; Malafouris 2008, 2009, 2010) and is a hallmark of humans’ ability to adapt to different environments. In fact, technological changes in the early Stone Age have been argued to coincide with evolving hominin brain size and language development, leading Malafouris (2009) to claim that “human brains and technology have been co-evolving for at least 2-million-years”—with neural plasticity arguably constituting a necessary prerequisite for this process. Therefore, it is possible that neural plasticity may have contributed to supporting behavioral and cognitive flexibility in terms of cultural learning, including tool use—whose production is itself a form of cultural learning.

**Figure 8.**
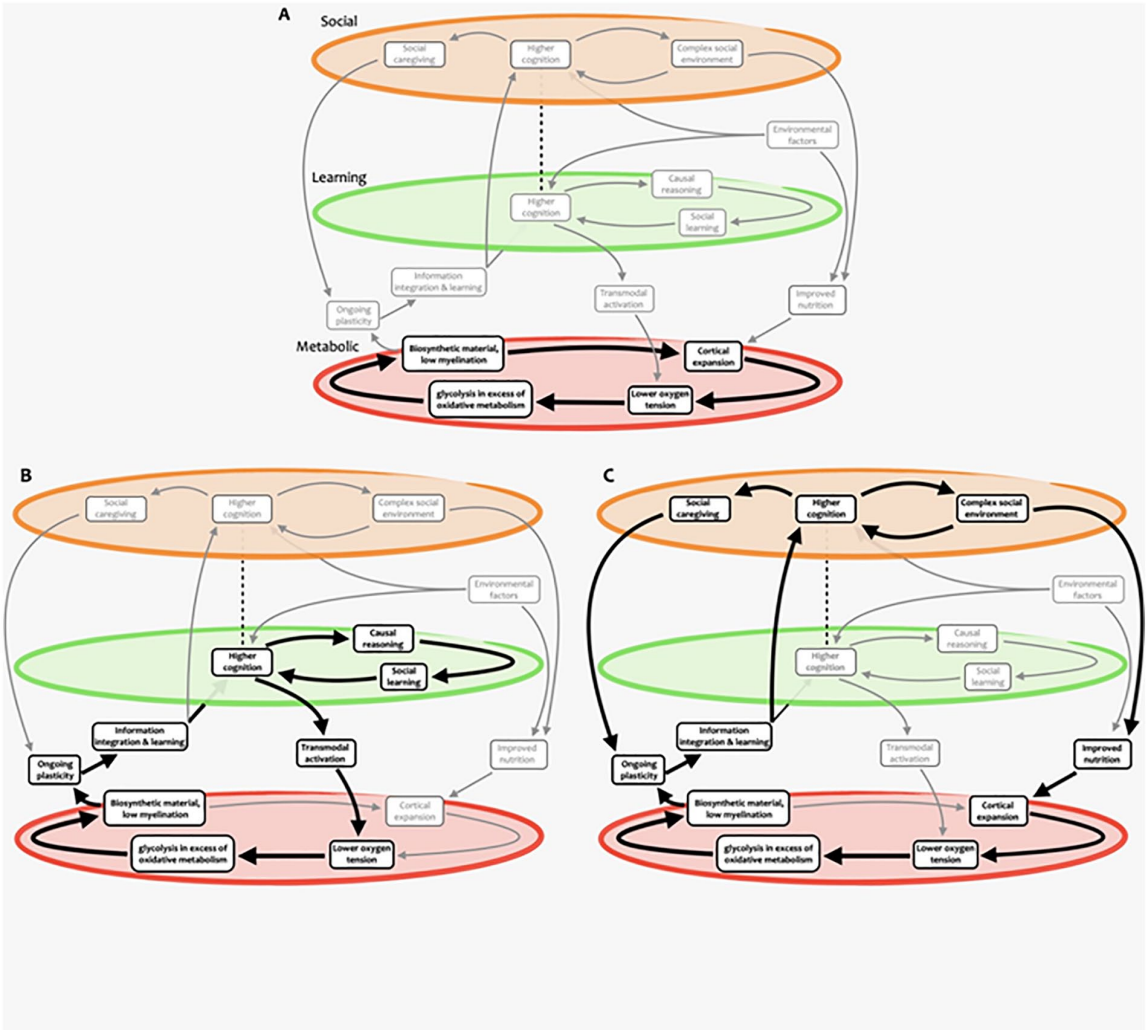
Nested self-reinforcing feedback loops supporting human brain expansion. Different cycles and subcycles are highlighted, including ones involving (A) metabolic, (B) learning and cognitive, and (C) social aspects. Note how some elements participate in multiple cycles at different scales. While these paths are highlighted for illustration purposes, we emphasize that more paths through this complex system could be devised, possibly representing hypotheses yet to be formulated and tested.

In summary, we build on the social brain hypothesis to argue that cooperating as part of a group provides greater capacity to obtain resources, while being able to form alliances and outmaneuver rivals in the group ensures a greater share of such resources for oneself and one’s offspring. Through this arms-race dynamic, the social environment has the potential for becoming continuously more complex as each generation’s skills at group politics and navigating social interactions grow, presenting a scenario wherein increased ability to negotiate with and benefit from others can keep getting advantageous. For this reason, we conjecture the existence of a feedback cycle between brain plasticity within the social brain areas and more complex social dynamics (Figure 8).

Indeed, there is within-species evidence in humans and rhesus macaques indicating that expansion of the cortical regions related to social cognition, which in humans are among the most evolutionarily expanded, covaries with social group size and may be brought about by increased social complexity. For example, early work from Bickart and colleagues (2011) reported that the size of a person’s social group, as indicated by the number of their contacts on the social network Facebook, correlated with thickness in prefrontal cortex and anterior cingulate cortex. The size of human DMN regions (gray matter volume) is also predicted by an individual’s skill at theory-of-mind tasks (Powell and others 2014; Powell and others 2010), which itself correlates with number of friends (Powell and others 2012; Stiller and Dunbar 2007), thereby offering connections among group size, social cognition, and size of the corresponding brain regions. Analogous results extend to other primates (Devaine and others 2017), which additionally provide causal evidence supporting that social network size influences regional gray matter. For example, Sallet and colleagues (2011) housed macaques in groups of different size and found that animals housed in larger social groups exhibited increased gray matter in the prefrontal cortex and superior temporal sulcus—regions of the macaque’s “social brain.” Additionally, the thickness of these regions was positively correlated with the animal’s social status within the group (Noonan and others 2014). More recently, an association was observed across primates between the number of cortical neurons and social complexity (indexed by size of the social group; Yokoyama and others 2021), providing converging evidence not only within but also across species.^16^ Taken together, these results provide evidence for a link between the opportunity and need for greater skill at social cognition and the cortical size of regions involved in it.^17^

### A Second Self-Reinforcing Cycle: Metabolism, Expansion, and Cognition

In turn, our metabolic/biosynthetic lens provides another key insight about the relationship between cortical expansion and cognitive demand. In addition to the cortical midline structures of the DMN, transmodal association cortices include lateral frontal and parietal cortices, broadly part of the so-called frontoparietal control network. Whereas the DMN is recruited by social cognition, as well as other situations that require transcending the here-and-now, the frontoparietal control network is engaged by cognitively demanding tasks, such as focused attention or working memory (Duncan 2010; Zanto and Gazzaley 2013) but also cognitive control and behavioral inhibition.^18^ The latter may be especially important for living in a social group: without the ability to inhibit prepotent responses, individuals would constantly be at risk of violating social norms and foregoing the advantages of group membership whenever a small but immediate reward comes within reach (Dunbar 2018).

Because DMN and frontoparietal control network activation is typically inferred from functional MRI, this means that the most expanded regions are also those that most increase their consumption of oxygen and glucose in response to social engagement or cognitive demand. Importantly, these regions are also those where glucose metabolism most exceeds the availability of oxygen for oxidative metabolism. Thus, although activation (e.g., by engaging in social cognition or a difficult task) leads to more oxygen being brought to these regions, we argue that their metabolic demand exceeds this delivery of oxygen, meaning that they would need to rely on GEOA to an even greater extent than usual to extract the required energy from glucose. Indeed, evidence indicates that in instances of acute metabolic demand such as functional activations, the expansion of oxygen consumption is quite limited and is outstripped by greater glucose delivery, sometimes in excess of the metabolic demand (Paulson and others 2010).

As we argue in the first part of this article, oxygen is more than just fuel for neuronal activity: GEOA generates biosynthetic materials as a by-product. As a result, by further increasing GEOA in evolutionarily expanded association cortices, engagement in social interactions and cognitively demanding activities should increase in these regions the availability of biosynthetic materials that are required for synapse formation and turnover. Taken together, in the context of our proposed account, this means that engaging with a socially rich and challenging environment provides 1) an occasion to learn from complex and challenging experiences and other beings and 2) increased availability of biosynthetic material to support this learning at the neural level. Crucially, this availability occurs in the regions that are most plastic, are involved with integrating multimodal information, and play a prominent role in supporting social cognition and cognitive control.

Therefore, to benefit from the opportunities that it offers, the individual will need ongoing capacity for learning: that is, ongoing plasticity. As we have argued, this is precisely what is available in evolutionarily expanded association cortices, which exhibit:

- A high capacity to integrate multimodal information
- Transcription of genes pertaining to synapse formation and low levels of plasticity-inhibiting intracortical myelination
- The biosynthetic material required to enact synapse formation and turnover, made available as a by-product of GEOA.

In turn, a self-reinforcing cycle exists between learning and evolution: although what is learned by the phenotype cannot be transmitted to the genotype, the capacity for flexible learning can provide a path for evolution in environments where no viable evolutionary path is available for nonlearning organisms (Hinton and Nowlan 1996). Indeed, computational work has shown that for complex evolutionary searches, this fact can provide a considerable boost to evolutionary time scales because a learning trial is enormously less time- and energy-consuming than the production and evaluation of an entire de novo organism (Hinton and Nowlan 1996). The invention of cooking and its proposed role in overcoming the energetic barrier to neuron count and brain expansion (Fonseca-Azevedo and Herculano-Houzel 2012) may be seen as a prominent example of evolution being shaped by learning. Intergenerational learning thanks to group life and language provides humans with a further multiplier of this powerful adaptation (Boyd and others 2011).

### The Broader Cycle: Expansion and Evolution

Overall, the account outlined so far provides converging reasons to explain why networks of transmodal association cortices would have been the focus of selective pressure. First, these cortices support ongoing learning—from one’s own experience and others. Second, they enable the individual to navigate a complex social environment. In turn, our oxygen-focused perspective provides a neurobiological account of how a socially and cognitively enriched environment contributes to a differential development of the two ends of the cortical processing hierarchy: activity in response to social and cognitive demand increases GEOA at the higher (associative) end of the cortical hierarchy—making available additional biosynthetic material and contributing to plasticity and ongoing learning.

Importantly, the prolonged period of immaturity that accompanies the development of a highly plastic brain is made affordable by being part of a group, which can provide essential protection for juveniles from predators. Additionally, being part of a group that hunts and gathers food together provides access to more diverse and higher-quality nutrition, which is required to fuel high cortical metabolism and enable cortical expansion. Finally, being part of a group provides ample opportunity for intergenerational learning and for acquiring and practicing social cognition skills. The conjunction of all these elements sets a broader self-reinforcing cycle that brings together brain expansion, cognition, and evolution, constituting the core of our account (Figure 1).^19^

## Conclusion and Future Directions

GEOA was recently dubbed “a mystery in neuroscience” (Theriault and others 2021). In this article, we propose to turn this statement on its head and use GEOA to make sense of another mystery: the evolution of the human brain. The account sketched here proposes that the apparent uniqueness of humans’ cognitive capacities may be best understood as emerging from nested, self-, and mutually reinforcing cycles involving several factors—metabolic, molecular, and behavioral—in the context of a favorable environment. Correspondingly, this view posits that what is unique to humans might be not any of these individual factors but rather the unique interaction among the three.

Under this perspective, metabolic fixation of O_2_ on carbon generates energy and a tendency toward growth. In the case of the human cortex, our account posits that the expansion of neocortical regions went beyond what is expected from allometric growth, outstripping vascular efficiency and making these cortices reliant, in part, on GEOA as a metabolic pathway, which generated conditions for protracted plasticity. Our account proposes that this in turn enables the integration of complex information and continuous learning, supporting greater flexibility in response to the environment and its cognitive challenges and paving the way for a rich social environment. A socially complex and cognitively stimulating environment provides the opportunity for ongoing learning and cognitive flexibility to become advantageous, while requiring greater activation of the association cortices subserving high-order cognition, resulting in even greater GEOA in these regions and, as a by-product, increased availability of biosynthetic materials for synaptic growth and turnover. Additionally, a more complex social organization affords several benefits, including protection during a protracted development period, intergenerational learning, and improved nutrition to fuel the metabolic demands of plastic expanded cortices.

The account presented here may be relevant for the framing of further studies of neurodevelopmental disorders and mental health. As a first example, metabolism and vascular health are already well-known factors underpinning psychiatric disorders (Baruah and Vasudevan 2019; Meier and others 2013; Sukumar and others 2020; Turkheimer and others 2020). By emphasizing the role of metabolism in controlling growth and cellular composition during brain maturation (Iwata and others 2021), this model points to a need for imaging technologies able to assay blood flow as well as oxygen and glucose metabolic rates in very young cohorts. Hence, experimental metabolic modulation should be added to current advanced organoid research (Notaras and others 2021) to illustrate how metabolic deficits may precipitate genetic risks for mental or degenerative disorders or how enhanced metabolism may recover genetic phenotypes. There is also evidence that respiratory complications and hypoxia are associated with increased rates of psychosis (Kalucy and others 2013; Partti and others 2015), which can also be induced acutely by extreme altitude (Hüfner and others 2018)—pointing to further promising lines of investigation about natural or induced variations in oxygen supply to the brain. Metabolic therapies as well as hyperbaric treatments have shown very mixed results in the treatment of mental illness in the young (Cheng and others 2017; Rossignol and others 2007; Rossignol and others 2009), but imaging assays could be used to phenotype those young patients who may best respond to these treatments as well as to inform on the optimal timing of interventions along the developmental trajectory. Brain imaging could also provide potential insights into healthy versus pathologic aging at the other end of the life span.

As a second example, the mechanistic links between metabolism and cellular differentiation in development suggest that in those psychiatric disorders where there is evidence of disturbed oligodendrocyte function and myelin, not only in the developing brain—as in psychosis (Mighdoll and others 2015)—but also in the adult brain in major depression (Takahashi and others 2011; Williams and others 2019), the underlying pathophysiology may be driven by disordered tissue oxygenation. More broadly, consideration of an individual’s regional balance of oxygen/glucose metabolism may shed light on the opposite end of the neurodevelopmental trajectory (e.g., neurodegenerative disorders).

We also note that glycolysis is associated with cell growth and proliferation across a variety of contexts (Lunt and Vander Heiden 2011; Vander Heiden, and others 2009). Although fermentation of glucose into lactate is less energy-efficient than oxidation in the mitochondria, glycolysis allows up to a 100-fold faster rate of glucose metabolism than oxidative phosphorylation (Liberti and Locasale 2016; Pfeiffer and others 2001). Because many unicellular organisms rely on fast proliferation to outcompete other cells, the faster metabolic rate of glycolysis makes it an appealing metabolic strategy even in the presence of abundant oxygen (Lunt and Vander Heiden 2011). Such a strategy is unfortunately also adopted by malignant cells (Venneti and Thompson 2017); for example, cancer cells are notoriously glucose hungry and rely predominantly on glycolysis to metabolize the additional glucose that they consume (Lunt and Vander Heiden 2011). In fact, the propensity of tumors for glycolytic fermentation of glucose into lactate even when oxygen is available (known as the “Warburg effect”) was documented nearly a century ago (DeBerardinis and Chandel 2020; Liberti and Locasale 2016; Warburg 1924, 1956).

In light of this evidence, it is not surprising that recent investigations indicate a link between glycolysis and glioma. High-grade gliomas exhibit the Warburg effect (Cairns and others 2011; Koppenol and others 2011), with elevated glycolysis mirroring the rate of tumor proliferation (Vlassenko and others 2015) and a recent report indicating that elevated expression of glycolytic genes corresponds to lower survival in patients with high-grade glioma (Stanke and others 2021). Additionally, evidence from a large sample of patients indicates that gliomas tend to occur most commonly in the temporal and especially frontal association cortices (Mandal and others 2020). Thus, although here we focus on the effects of GEOA in the context of healthy brain development and cortical expansion, the same metabolic process can underpin pathologic runaway proliferation.

Intriguingly, a recent study indicated that synapse-rich regions of high metabolic activity are particularly vulnerable to inflammation associated with depression, due to permeability of the barriers (Althubaity and others 2022). Likewise, growing evidence indicates that brain regions with more recent evolutionary changes may be more susceptible to pathologies such as Alzheimer’s disease than more evolutionarily stable regions (Bruner and Jacobs 2013). Additionally, glycolysis is the dominant metabolic pathway in proinflammatory cells (Pearce and Pearce 2013). In turn, neuroinflammation has been shown to increase accumulation of iron in neurons and microglia, as indicated by atomic absorption spectroscopy (Ward and others 2014). Given its capacity for reaction with oxygen, iron is a key element in oxygen-involving processes in the brain, including oxygen transportation, oxidative phosphorylation, neurotransmitter synthesis and metabolism, and myelin production in oligodendrocytes (Ward and others 2014). However, the same reactivity makes it liable to induce oxidative stress as a result of generating reactive oxygen species. By favoring inflammation, which promotes iron accumulation, GEOA may be leading to the accumulation of iron in the regions that least require it: association cortices, which are low in myelination and oxidative phosphorylation, two of the most prominent consumers of iron in the brain. This unused iron would then be left to induce oxidative stress in association cortices, possibly contributing to their vulnerability and neurodegeneration. These observations raise the additional question of how the human brain balances the costs and benefits of GEOA.

By embracing complexity, we acknowledge that the account presented here is inevitably incomplete: not only could there be additional paths connecting the various elements of Figure 1 (as outlined in Figure 8), but additional elements will almost certainly need to be considered to provide the full picture. We also note that we have considered here only the effects of circulation on oxygen and nutrients delivery; we have not addressed energy dissipation/cooling and the associated thermal distribution, which will need to be integrated (Bruner and others 2012; Karbowski 2009). Likewise, we have not considered CSF circulation and the effects that cortical expansion may have exerted on the glymphatic system (Jessen and others 2015). Additionally, we acknowledge that the account provided here has been decidedly “cortico-centric” and that extending this account to subcortical structures will be an important next step (Barger and others 2014). Of particular interest for future developments is the role of the cerebellum: the cerebellum displays one of the lowest rates of GEOA, yet ultrahigh-resolution neuroanatomic investigation of human and monkey specimens suggests that the human cerebellum has expanded its surface area even more than the cerebral cortex (Sereno and others 2020). Abnormal cerebellar function is also associated with a multitude of psychiatric and neurodegenerative disorders (Argyropoulos and others 2020; Gellersen and others 2017; Gellersen and others 2021; Moberget and Ivry 2019), and prefrontal-projecting cerebellar lobules are notably expanded in humans relative to other primates, suggesting potential interplay between cerebellar and cortical expansion (Balsters and others 2010). Similarly, evidence for evolutionary expansion of limbic structures including subcortical ones (hippocampus, amygdala; Barger and others 2014) is currently not included in the account presented here and will represent an important avenue for future additions. Finally, we emphasize that not all the evidence presented here is direct, providing an opportunity for specific empirical testing of our hypothesis, and more broadly the role of neurovascular coupling remains largely mysterious, as outlined in a recent review (Drew 2022) that identified several potential and possibly complementary roles beyond the supply of oxygen.

Overall our proposal emphasizes the relevance of oxygen and metabolism on brain evolution and highlights the benefits of a complex-systems approach for integrating different perspectives about brain evolution, highlighting their complementarity and interdependence. It is our hope that this work may foster future investigations shedding further light to these promising avenues for deepening our understanding of what makes human brains unique.

## Notes

1. Allometry is the study of the relationship of body size to shape, anatomy, physiology, and finally behavior. Allometric scaling relations—for example, the 3/4 power law scaling for metabolic rates with body mass, also known as Kleiber’s law—are characteristic of all organisms (West and others 1997). These relations are based on the general principle that cellular metabolism requires essential nutrients and importantly oxygen, which are transported through space-filling fractal networks of branching tubes.

2. The prefrontal cortex is a region of particular interest due to its role in a range of specialized cognitive capacities, such as language, imagination, and complex decision making (Gabrieli and others 1998; Miller and Cohen 2001; Rushworth and others 2011). The granular prefrontal cortex may even be unique to primates (Preuss 1995; Striedter 2004). Because the prefrontal cortex and associative cortices do not scale well across anthropoid primates, their expansion is now purported as a nonallometrically derived feature of cortical organization (Sherwood and Smaers 2013; Smaers and others 2017).

3. Such organoid systems remain experimental models and are not a replacement for actual brain experimentation. With this caveat in mind, data from such systems are extremely useful because they permit ethical access to, and precise control of, human cells in a 3-dimensional environment that may be considered, very broadly, similar to early brain development.

4. Given its separation from aqueous vapor in the atmosphere by ultraviolet radiation from the sun (Lu and others 2014, Nisbet and Sleep 2001). This was even before its rapid increase due to the advent of oxygenic photosynthesis during the Proterozoic aeon of the Precambrian period, ∼2500 to 540 million years ago.

5. See Bernier and others (2018) for a different atlas obtained at lower resolution (3 T).

6. Note that there is considerable heterogeneity in oxygen levels within the body (21% O_2_ in the atmosphere, 14% O_2_ in the alveolar air, 12% O_2_ in the arterial blood, and 5.3% O_2_ in venous blood), with a mean of 3% O_2_ in tissues. In the mammalian brain, interstitial tissue oxygen levels are low and nonuniform, ranging from ∼1% to 5% (Lahiri and others 2006).

7. This process is typically referred to as “aerobic glycolysis.” Nonetheless, in this article, we avoid this terminology because of the potential to create confusion, because our argument is that this process occurs due to insufficient oxygen being available, as outlined later.

8. In the human brain, GEOA at the regional level is revealed by in vivo PET scanning with radiolabeled glucose and oxygen to estimate a molar ratio between their respective metabolic consumption: if all glucose is metabolized through oxidative phosphorylation to produce CO_2_ and H_2_O, then a 6:1 ratio should be observed between oxygen consumption and glucose metabolism, whereas a lower ratio indicates that some of the glucose is not being oxidized (Vaishnavi and others 2010).

9. We note that mild hypoxia does not seem to diminish ATP levels but does substantially decrease the turnover of several neurotransmitters (Gibson, Pulsinelli, and others 1981). Because many neurotransmitters have oxygen-limited synthesis pathways (Drew 2022; Gibson and Duffy 1981; Gibson, Peterson, and Sansone 1981; Goldstein and others 1968), the observations of reduced neurotransmitter turnover but preserved ATP levels during hypoxia together suggest that an alternative source of energy that does not rely on oxygen (ie., glucose to lactate) steps in while glucose metabolism alone cannot sustain the bulk of energy budget that supports neurotransmitter turnover. Additionally, glycolysis is performed by not only neurons but also astrocytes (Figure 4), which release lactate that is shuffled to neurons, converted into pyruvate, and then oxidized (Hertz and Rothman 2017). Interestingly, recent transcriptomic evidence (Medel and others 2022) indicates that the *MCT2* gene that codes for the lactate shuttle is reduced from primary cortices to association cortices. This observation could be explained if association cortices were, per our account, limited in terms of their oxygen tension such that no residual oxygen would be available to oxidize the extra lactate coming from the astrocytes: in such a scenario, there would be no point in transporting lactate into neurons that cannot metabolize it.

10. A variety of meanings of “cortical hierarchy” exist (Hilgetag and Goulas 2020); here we refer to the brain’s sensory-fugal organization as recapitulated by molecular, functional, and anatomic regional heterogeneity (Burt and others 2018; Hansen and others 2021; Luppi and others 2022; Margulies and others 2016).

11. This account is not intended to be the sole explanation for regional differences in cytoarchitecture: indeed, transcriptomic data indicate that the expression of genes promoting different cell types is also regionally heterogeneous, with oligodendrocyte-related genes being preferentially expressed in primary cortices, as expected from their elevated levels of intracortical myelination (Burt and others 2018). Therefore, our account proposes that genetic developmental programs and differences in tissue metabolism converge to produce interregional differences in cytoarchitecture.

12. Technically, neoteny refers to a change of size (growth) without a change of shape (development).

13. Oxygen and metabolism play more than one role in neurodevelopment: evidence from rodents indicates that reactive oxygen species mediate adult hippocampal neurogenesis in response to physical activity (Adusumilli and others 2021). Conversely, proliferation and neurogenesis of hippocampal neural stem/progenitor cells of the mouse are impaired by a genetic mutation that compromises lipid metabolism, leading to cognitive deficits; furthermore, embryonically derived cerebral organoids showed reduced proliferation, possibly implicating impaired prenatal neurogenesis in the humans with the mutation (Bowers and others 2020). See Namba and others (2021) for a recent review of metabolic regulation of cortical development and evolution.

14. We emphasize that the socially focused account outlined here should be understood as a “minimal working example” rather than a definitive explanation encompassing all possible relevant factors. For instance, physical exercise influences the extent and spatial distribution of brain oxygenation in rodents (Lu and others 2020) and is also known to induce neurogenesis in rodents (Overall and others 2013). In humans, cross-sectional and interventional evidence associated aerobic exercise in older adults with greater cortical and whole brain volume, as well as increased gray matter volume of the lateral temporal and prefrontal cortices (Raichlen and Alexander 2014). It has been argued that the shift to a hunter–gatherer diet imposed increasing demands for endurance running on *Homo erectus* (Raichlen and Alexander 2014). Therefore, this increase in physical activity and its effect on regional oxygenation may have played a role in the oxygen-dependent expansion of human association cortices—a hypothesis that warrants further investigation, especially given the well-established positive effects of physical exercise on mental health and cognitive aging (Raichlen and Alexander 2014).

15. Also known as the “Machiavellian intelligence hypothesis.” This line of reasoning has been extended by the more recent “Napoleonic intelligence hypothesis” (Ashton and others 2020).

16. Additionally, the social environment is known to be particularly salient during adolescence, when higher-order regions of the “social brain” are still maturing, which may account for further interdependencies between the two (Sydnor and others 2021).

17. To be clear, this evidence pertains to changes within the life span rather than across evolution, and it does not tell us whether such association was in fact selected over evolutionary time scales—although this account is compatible with the evolutionary expansion of the same regions. Nevertheless, it does tell us that a more complex social environment may directly promote the expansion of these regions in the primate brain.

18. Note that the distinction is not clear-cut: see Vatansever and others (2015a, 2015b) for evidence of DMN involvement in cognitive tasks and Jackson and others (2022) for evidence suggesting frontoparietal control network rather than DMN involvement in social cognition.

19. Needless to say, our account is inevitably an oversimplification of the immensely rich and complex web of interactions among metabolism, expansion, social cognition, and evolution. As just one example, under conditions of maternal immune activation, resources and energy are switched in the fetal brain to protection from inflammatory insult, and recent evidence indicates that mice exposed in utero to maternal immune activation have a low social preference index, which may be the strongest, though not the only, predictor of susceptibility (Mueller and others 2021). In the frontal cortex of these susceptible animals, there is also down-regulation of mitochondrial and metabolic pathways. Thus, the rich interplays between these aspects are not confined to the evolutionary scale but rather occur across diverse time scales.
